# Independent control of harmonic amplitudes and phases via a time-domain digital coding metasurface

**DOI:** 10.1038/s41377-018-0092-z

**Published:** 2018-11-21

**Authors:** Jun Yan Dai, Jie Zhao, Qiang Cheng, Tie Jun Cui

**Affiliations:** 1 State Key Laboratory of Millimeter Waves, Southeast University, 210096 Nanjing, China; 2 Synergetic Innovation Center of Wireless Communication Technology, Southeast University, 210096 Nanjing, China

## Abstract

Harmonic manipulations are important for applications such as wireless communications, radar detection and biological monitoring. A general approach to tailor the harmonics involves the use of additional amplifiers and phase shifters for the precise control of harmonic amplitudes and phases after the mixing process; however, this approach leads to issues of high cost and system integration. Metasurfaces composed of a periodic array of subwavelength resonators provide additional degrees of freedom to realize customized responses to incident light and highlight the possibility for nonlinear control by taking advantage of time-domain properties. Here, we designed and experimentally characterized a reflective time-domain digital coding metasurface, with independent control of the harmonic amplitude and phase. As the reflection coefficient is dynamically modulated in a predefined way, a large conversion rate is observed from the carrier signal to the harmonic components, with magnitudes and phases that can be accurately and separately engineered. In addition, by encoding the reflection phases of the meta-atoms, beam scanning for multiple harmonics can be implemented via different digital coding sequences, thus removing the need for intricate phase-shift networks. This work paves the way for efficient harmonic control for applications in communications, radar, and related areas.

## Metasurfaces: Reflecting radiation in harmony

A reflective metasurface allows researchers in China to control the harmonic frequencies present in electromagnetic radiation. Artificial metasurfaces contain periodic arrays of resonating structures called meta-atoms, which can manipulate light and other radiations because they are smaller than the incident wavelengths. Tie Jun Cui and co-workers at Southeast University in Nanjing have developed a metasurface where each meta-atom contains a varactor diode – an electrical component whose capacitance varies with voltage. They can therefore use voltages to control the phases and amplitudes of microwave harmonics that are reflected by each diode. The team went on to encode an array of 56 diodes to produce multiple harmonics at once. In future, this design could operate on shorter wavelengths such as visible light by scaling down the size of the meta-atoms, benefiting radar and communications.

## Introduction

Metasurfaces provide an unprecedented route to control light propagation and engineer light-matter interaction using an array of resonators with carefully designed geometries. With the introduction of an abrupt phase discontinuity along the interface, the electromagnetic (EM) properties of incident light can be tailored in a controlled manner as governed by the generalized Snell’s law, resulting in full control of the propagation direction, polarization, and wavefront shape over subwavelength distances. These distinctive features have driven a rich variety of new technologies such as holographic imaging, perfect absorption and nonreciprocal transmission^1–11^. With the rapid advent of various tuning technologies, a number of tunable metasurfaces have been shown to provide in situ dynamic control of electromagnetic waves, which opens a new route for a variety of applications such as beam shaping, active focusing and biosensing^12–18^.

However, one challenging task for the construction of a tunable metasurface involves the design of an intricate feeding network that provides various excitation intensities and phases to each element with high precision. In this regard, an alternative method has been explored, namely, coding the metasurface and digital metasurface, which shapes the phase front of the electromagnetic waves using binary meta-atoms with carefully designed geometries^19–24^. Such a configuration is extremely helpful to simplify the feeding network since only the discrete ON or OFF states for the modulation are required to manipulate the radiation or scattering properties of electromagnetic waves^25–29^.

In addition to engineering the spectral response with a space-gradient metasurface, the time discontinuities of the element phase provide an additional degree of freedom to control the normal momentum component at the interface of the time-varying metasurface, leading to a break in Lorentz reciprocity during the light-matter interactions without the use of nonlinear materials^30–39^. Due to the rapid growth of communication technologies, the time-modulated antenna was developed to radiate harmonics with tight control of wave behavior so that information could be delivered through multichannels^40–44^. However, such an antenna lacks the ability to modulate incoming waves with efficient harmonic manipulation.

Here, we report on a scheme to achieve individual control of the amplitude and phase for harmonics via a reflective time-domain digital coding metasurface. As the reflection phase of the meta-atom is periodically switched between two states, a series of harmonics emerges, with their intensities determined by the phase difference within the switching function. Meanwhile, extra time delays are imposed on the modulation signal of the meta-atoms to create a metasurface with nonuniform harmonic phase distributions, thereby enabling wavefront reshaping for high-order harmonics. When discrete phase states of the meta-atoms are encoded into the binary sequences, control of multiple harmonics including the beam directions and intensities is realized using elaborate coding strategies. All measured signals agree closely with the analytical predictions, with these unique features likely to have key roles in future radar and communication systems.

## Results

### Design of the metasurface

**Fig. 1:**
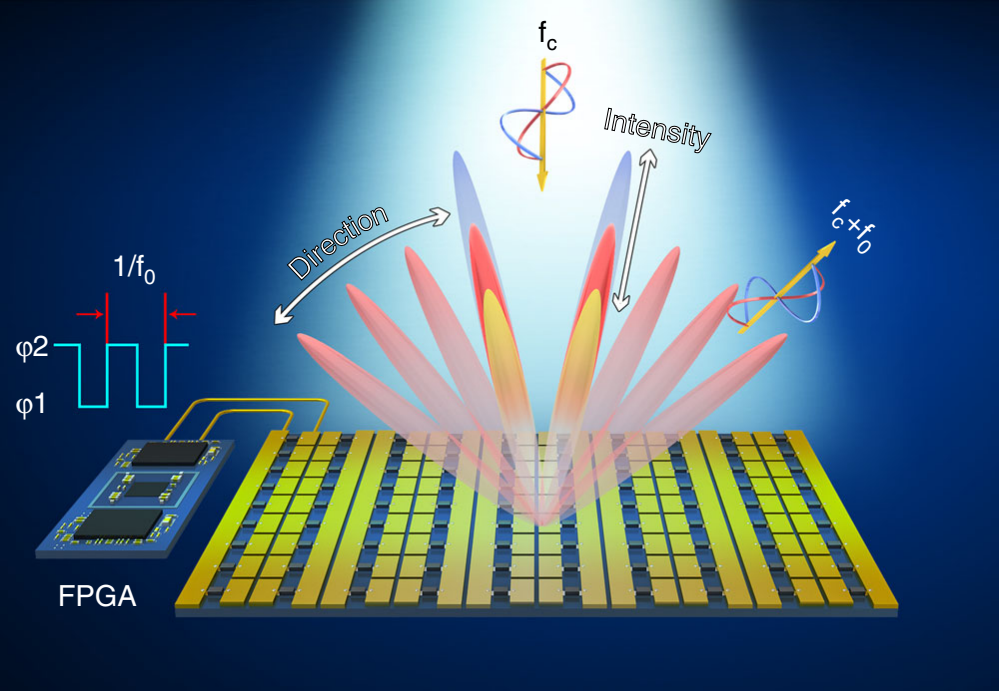
Schematic diagram of the time-domain digital coding metasurface, which is able to generate nonlinear harmonics under excitation by a monochromatic wave as well as control their direction and magnitude

**Fig. 2:**
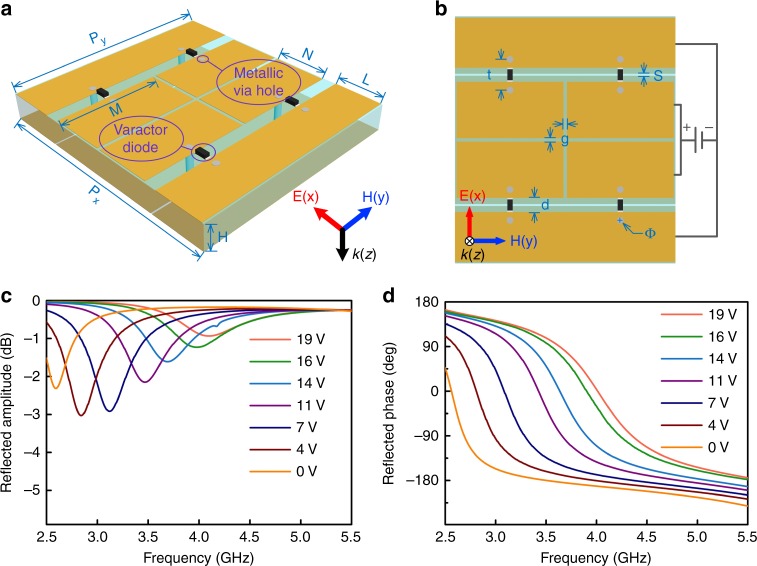
** Illustration of the time-domain digital coding meta-atom. a**, **b** Perspective and front views of the time-domain digital coding meta-atom. **c**, **d** The simulated reflection amplitude (**c**) and phase (**d**) spectra under different bias voltages. The dimensions of the meta-atom are *P*_x_ = 37 mm, *P*_y_ = 33 mm, *H* = 4 mm, *M* = 16 mm, *N* = 8.45 mm, *L* = 7.45 mm, *g* = 0.5 mm, *d* = 2.1 mm, *t* = 4.6 mm, Φ = 1 mm, *s* = 0.3 mm

We designed a reflective time-domain digital coding metasurface (Fig. 1) with each unit loaded with varactor diodes. The phase response of the meta-atom can be accurately tailored within a wide phase range (~270°) by tuning the biasing voltage of the diode. This can be easily implemented when the DC feed of the element is connected to the peripheral circuit responsible for the generation of the desired control signal level, as will be discussed later. A three-dimensional schematic view of the meta-atom is shown in Fig. 2a. The side and central rectangular patches of the proposed element are bridged by four varactor diodes in total. A slotted copper layer lies at the bottom of the element to act as the DC feeding network, where a biasing voltage is applied to the diode via holes. A more detailed element description is provided in Fig. 2b, where *P*_x_ = 37 mm, *P*_y_ = 33 mm, *H* = 4 mm, *M* = 16 mm, *N* = 8.45 mm, *L* = 7.45 mm, *g* = 0.5 mm, *d* = 2.1 mm, *t* = 4.6 mm, Φ (diameter of the hole) = 1 mm, and *s* = 0.3 mm. The top and bottom layers are spaced by a substrate (F4B) with a dielectric constant and loss tangent of 2.65 and 0.001, respectively. To prohibit wave transmission through the slots of the feeding layer, an extra copper layer is placed at the bottom of the metasurface, eliminating the possibility of energy leakage. In general, the varactor diode (SMV-2019, Skyworks, Inc.) can be regarded as an RLC model at the operation frequency with the equivalent circuit parameters listed in ref. ^45^. To grasp a complete picture of the reflection spectra for the meta-atom, a full wave simulation was performed using a commercial electromagnetic solver (CST Microwave Studio 2016) under different biasing voltages. The reflection minima was observed to redshift (Fig. 2c) as the biasing voltage was changed from 19 to 0 V. Good phase linearity and a large phase range near 3.7 GHz was observed, as shown in Fig. 2d. This enables beam steering based on the relative phase shift of adjacent elements similar to the principle of the phase array antenna. The layout of the metasurface consists of 7×8 elements (Fig. 2). For simplicity, all the varactor diodes in each column share the same biasing voltage. Targeting the discrete phases represented by the codes in our design, a series of biasing voltages, i.e., 0, 3, 6, 9, 12, 15, 18, and 21 V, were adopted to meet the phase demand in the experiment. The mapping relationship between the reflection phase and the biasing voltage for the meta-atom in the simulation is presented in Table 1, which provides a basis for the DC level on the diodes for each element in the following experiment.

**Table 1:** The mapping relationship between the reflection phase of the meta-atom and biasing voltage at 3.7 GHz in the simulation

Voltage (V)	0	3	6	9	12	15	18	21
Phase (Deg.)	0	10	30	90	180	210	250	270

### Experimental results

**Fig. 3:**
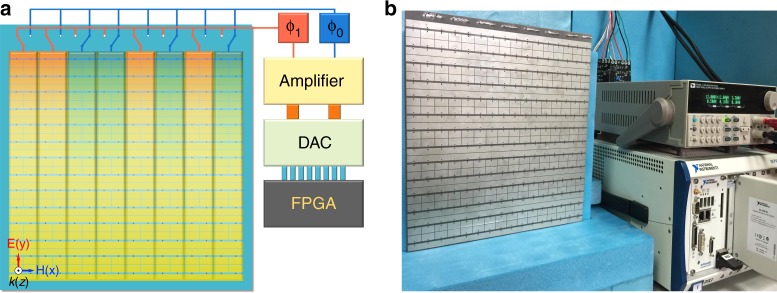
**Schematic and photograph of the time-domain digital coding metasurface. a** Systematic description of the time-domain digital coding metasurface. **b** Photograph of the fabricated sample (on the left) in the experiment. The control system is presented in the right of **b**, consisting of a DC power source, FPGA platform (NI Corp.), DAC module and an external amplifier module

During the experiment, two horn antennae were used to illuminate the sample and receive the reflected signal, respectively. Both the microwave signal generator and the spectrum analyzer were utilized to monitor the nonlinear properties of the metasurface over a broad spectrum when connected to the horn antennae separately through phase stable cables. The sample was fabricated using standard PCB technology, with all components described in Fig. 3a and b. A FPGA controller, a digital-analog conversion (DAC) module and an analog amplifier module were employed to ensure a high output swing in accordance with the large range of regulation voltages used for the selected varactors. The operation frequency *f*_0_ was 3.7 GHz and the control circuit was programmed to generate square wave control signals with different voltages, periods and time delays.

**Fig. 4:**
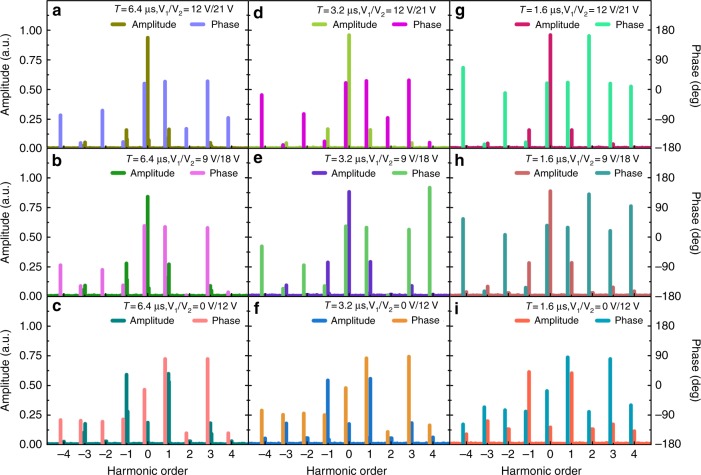
The measured harmonic intensities/phases distribution of the time-domain digital coding metasurface at 3.7 GHz, where different biasing voltages *V*_1_/*V*_2_ and modulation periods *T* are considered. The same modulation period *T* = 6.4 μs (**a**–**c**), *T* = 3.2 μs (**d**–**f**) and *T* = 1.6 μs (**g**–**i**) are applied in each column, corresponding to a frequency interval of 156.25 kHz, 312.5 kHz and 625 kHz, while the same biasing voltage *V*_1_/*V*_2_ = 12 V/21 V (**a**–**g**),*V*_1_/*V*_2_ = 9 V/18 V (**b**–**h**),*V*_1_/*V*_2_ = 0 V/12 V (**c**–**i**) is adopted in each row, respectively

Prior to measurement of the scattering patterns of the harmonics, it was important to observe the nonlinear generation capability of the sample through experiments (see theory in Materials and methods). Using Table 1, which relates the biasing voltage to the reflection phase, it is easy to identify the combination of biasing voltages *V*_1_/*V*_2_ corresponding to the required phases *φ*_1_ and *φ*_2_ in Eq. 4. During the experiment, we used a spectrum analyzer to directly measure the harmonic intensities. The harmonic phases were obtained through Fast Fourier Transformation of the echo signal from the metasurface, which was recorded by a software-defined radio reconfigurable device (USRP-2943, National Instruments Corp.). Figure 4 illustrates the relationship between the measured harmonic intensities**/**phases and the biasing voltage *V*_1_/*V*_2_ as well as the modulation period *T* when all the varactor diodes of the metasurface are biased with the same control signal.

Although the measured results in Fig. 4 indicate that the mapping relationship between the biasing voltage and reflection phase is not entirely consistent with the designs in Table 1, the nonlinear phenomenon still occurs. Consistent with theoretical predictions, the measured results clearly reproduce the trend of growing harmonics as *φ*_1_−*φ*_2_ approaches *π*. In contrast, the intensity of the synchronous component experiences a significant drop as most of the reflection energy is shifted into the harmonic channels, which is expected to be totally eradicated in the case of opposite phases for *φ*_1_ and *φ*_2_ from Eq. (8). Such a discrepancy may be ascribed to phase deviations of the meta-atoms from their ideal values under different biasing voltages, largely stemming from the parasitic parameters of the varactor diode, the fabrication tolerance and distortion of the control signal. The last issue is caused by the small switching time of the biasing signal that is beyond the bandwidth of the control circuit, which in turn damages the edges of the square waveform.

Figure 4 reveals that the modulation period has nearly no influence on the harmonic intensity but plays a vital role in determining the positions of the spectral lines. With a decrease of the modulation period from 6.4 to 1.6 μs, the observed frequency gap between adjacent harmonics is increased by up to 625 kHz in the experiment, proportional to 1/T. In addition to controlling the spectral position of the first harmonic, the possibility of amplitude modulation with the metasurface is shown in Fig. 4. For example, the amplitude of the +1^st^ order harmonic is closely associated with the biasing voltage combination *V*_1_ and *V*_2_ from each column in Fig. 4. Therefore, the task of amplitude modulation can be easily implemented by tuning the external biasing voltages.

The designed metasurface offers a wide dynamic range of up to 25 dB for the +1^st^ order harmonic amplitude with respect to different voltage combinations. This is ideally suited for accurate tuning of the harmonic intensity in practice. For better demonstration in the following experiment, three sets of parameters with 0 V/12 V (*A*_1_), 9 V/18 V (*A*_2_), and 12 V/21 V (*A*_3_) were picked up manually to meet the requirement for amplitude attenuation by 0, 5, and 10 dB for the +1^st^ order harmonic when reflected from the sample.

**Fig. 5:**
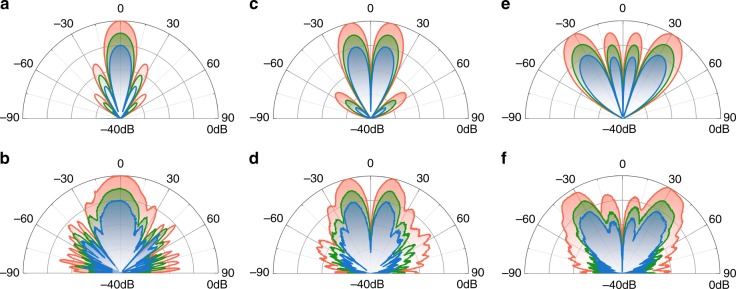
**Calculation and measurement results for the +1**^**st**^ **order harmonic of the metasurface.** The calculated (**a**, **c**, **e**) and measured (**b**, **d**, **f**) *E*-plane scattering patterns of the +1st order harmonic under different coding sequences of the metasurface. The first to third columns correspond to the coding sequences of ‘00000000’, ‘00001111’ and ‘00110011’, respectively. The red, green and blue lines in (**b**, **d**, **f**) demonstrate variance of the scattering magnitude due to changing the voltage combination from *A*_1_ to *A*_3_

We then experimentally investigated the modification of the scattering pattern of the +1^st^ order harmonic via the proposed metasurface. The modulation period was *T* = 6.4 μs, indicating that the +1^st^ order harmonic operates at a frequency of 3.70015625 GHz. Following the derivation in Eq. (8), the control signal shift in time *t*_0_ can lead to an extra phase lag *ω*_0_*t*_0_ for the +1^st^ order harmonic without altering its magnitude (see Materials and methods). Thus, the time delay 0(0 μs) and *T*/2(3.2 μs) can be employed respectively to represent the coded elements ‘0′ and ‘1’ with opposite phase. Consequently, the reflection phases of all the columns in the metasurface (Fig. 3a) can be described by the coding sequence of ‘0’ and ‘1’ bits. In case of the metasurface encoded by ‘00000000′ with no phase difference for all the columns, a directive scattering beam was measured for the +1^st^ order harmonic along the normal direction, as shown in Fig. 5b. The biasing voltages also yielded magnitude control for the scattering pattern without changing its profile. By varying the voltage combination from *A*_1_ to *A*_3_, a decay rate of 0, 5, and 10 dB was observed for the scattering magnitude from the red, green and blue lines in Fig. 5b. Further characterization of the scattering patterns for the codes ‘00001111’ and ‘00110011’ can be found in Fig. 5d–f, in which the main scattering lobe was beamed toward different angular directions via the interference from the columns, enabling a large range of magnitude adjustment via control of the biasing voltages similar to that in Fig. 5b. By comparing Fig. 5a–e and Fig. 5b–f, one can observe a good correspondence between the theoretically calculated and experimentally measured results. Such agreement suggests potential for the beam forming the +1^st^ order harmonic via the time-domain digital coding metasurface with sufficient accuracy.

**Fig. 6:**
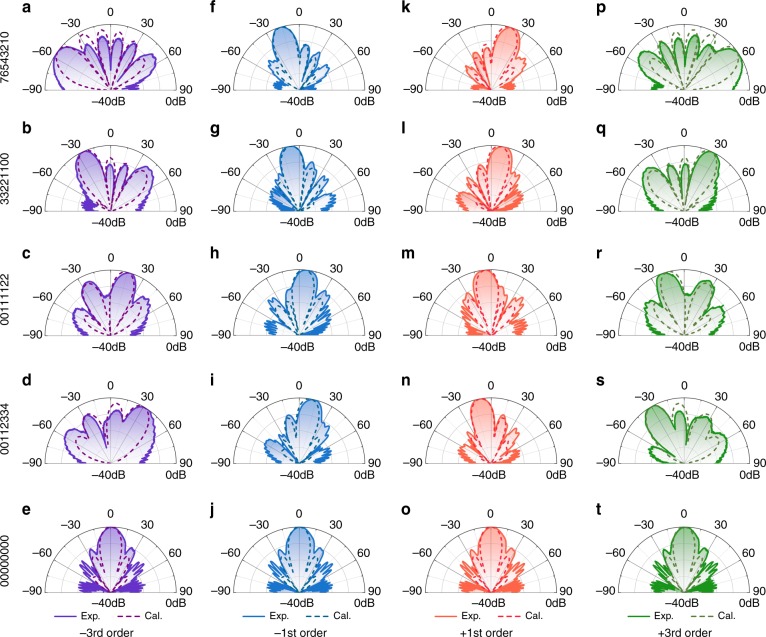
**The calculation and experimental results for multi order harmonics of the metasurface.** Calculated (dashed line) and experimental (solid line) E-plane scattering patterns for the −3^rd^ (purple lines), −1^st^ (blue lines), +1^st^ (red lines) and +3^rd^ (green lines) order harmonics under the different coding sequences shown on the left

Finally, we explored the feasibility of multiharmonic control with the same strategy. As mentioned above, the time delay *t*_0_ of the control signal introduced an additional phase shift *kω*_0_*t*_0_ to the harmonic of *k*^th^ order. This interesting property hints toward a novel route for controlling the scattering patterns of multiple harmonics at the same time. Here, we aimed to simultaneously tailor the scattering features of −3^rd^, −1^st^,  +1^st^, and  +3^rd^ order harmonics in experiment. To achieve a more flexible control of the harmonics, the 3-bit meta-atoms with eight phase states were adopted by applying various time delays t_0_ to the square wave functions. The codes ‘0’, ‘1’, ‘2’, ‘3’, ‘4’, ‘5’, ‘6’, ‘7’ were used to represent 0, *π*/4, *π*/2, 3*π*/4, *π*, 5*π*/4, 3*π*/2, 7*π*/4 for the +1^st^ order harmonic when *t*_0_ equals 0 (0 μs), *T*/8 (0.8 μs), *T*/4 (1.6 μs), 3 *T*/8 (2.4 μs), *T*/2 (3.2 μs), 5 *T*/8 (4 μs), 3 *T*/4 (4.8 μs), 7 *T*/8 (5.6 μs). However, the coding implications led to a great difference with changing harmonic order, which corresponds to (0, −*π*/4, −*π*/2, −3*π*/4, −*π*, −5*π*/4, −3*π*/2, −7*π*/4), (0, 3*π*/4, 3*π*/2, 9*π*/4, 3*π*, 15*π*/4, 9*π*/2, 21*π*/4) and (0, −3*π*/4, −3*π*/2, −9*π*/4, −3*π*, −15*π*/4, −9*π*/2, −21*π*/4) for −1^st^,  +3^rd^, and −3^rd^ order harmonics, respectively. This change means that these harmonics have different scattering orientations under the same coding sequences. The −3^rd^, −1^st^, +1^st^, and +3^rd^ order harmonics were set to operate at 3.69953125, 3.69984375, 3.70015625, and 3.70046875 GHz, respectively. We investigated the waveform modulation phenomena over the aforementioned four harmonics when switching among the predesigned coding sequences. Figure 6 demonstrates the measured E-plane scattering patterns of the −3^rd^ (purple line), −1^st^ (blue line),  +1^st^ (red line) and  +3^rd^ (green line) order harmonics monitored by the spectrum analyzer, along with the corresponding simulation results. Five coding schemes were applied to the eight columns of the metasurface from left to right (Fig. 3), including ‘00000000’, ‘00112334’, ‘00111122’, ‘33221100’, and ‘76543210’. The calculated results (dashed lines) show how the variance of the coding sequence affects the scattering response of the four harmonics under normal illumination by plane waves at 3.7 GHz, based on the Fourier transformation between the surface currents and the far fields^46,47^. Due to the linear relationship between the phase shift and the harmonic order, it was possible to achieve a larger deflection angle for high-order harmonics. This can be confirmed by comparing the scattering patterns with the same coding sequence in each line of Fig. 6. Moreover, symmetric scattering patterns could be found for the harmonic pairs (−1^st^,  +1^st^) and (−3^rd^,  +3^rd^) (Fig. 6) since opposite phase distributions were obtained with the same coding sequence, which thus gave rise to reversed in-plane momentum added to that of the incident wave. The close agreement between the measured and calculated scattering angles suggests that the time-domain digital coding metasurface can serve as a good candidate to realize a spatial scan with multiple harmonics, e.g., one can simultaneously scan the angular region from 0° to 90° by the  +1^st^ order harmonic and the region from −90° to 0° by the −1^st^ order harmonic, and thus reduce the time cost for measurement by half.

## Discussion

In this study, we created a time-domain digital coding metasurface to generate harmonics under the illumination of electromagnetic waves with a high conversion rate, which enables independent control of the harmonic amplitude and phase. By controlling the biasing voltages of the varactor diodes incorporated into the meta-atoms, it was possible to acquire a periodic time-variant reflection coefficient responsible for regulating the harmonic intensity. Meanwhile, the time delay of the switching functions for the meta-atoms can be carefully selected to modify the harmonic reflection phase, so that the metasurface can be employed to reshape the wavefront for multiple harmonics. Although our work was experimentally confirmed at microwave frequencies, it can be further extended into the THz and light regime in combination with advanced modulation technologies^32,33^. Furthermore, the transmission type metasurface can also be employed to realize harmonic manipulation when it is fed by a patch antenna array from behind.

## Materials and methods

### The nonlinear theory for the time-domain digital coding metasurface

We started by considering the reflection problem when EM waves are illuminated normally from the upper free space upon a reflective time-domain digital coding metasurface. The temporal expression of the reflected wave can be written as ${\mathrm{E}}_r(t) = \Gamma (t) \cdot {\mathrm{E}}_i(t)$, where ${\mathrm{E}}_r(t),{\mathrm{E}}_i(t)$ and $\Gamma (t)$ denote the reflected wave, incident wave, and reflection coefficient, respectively. Specifically, in the case of monochromic incidence at frequency *f*_*c*_ where ${\mathrm{E}}_i(t) = e^{ - j\omega _ct}$, the reflected wave in the frequency domain reads

1 $${\mathrm{E}}_r(\omega ) = \Gamma (\omega ) \ast \left[ {\delta \left( {\omega - \omega _c} \right)} \right] = \Gamma (\omega - \omega _c)$$

where *ω*_*c*_ the is angular frequency and *δ*(*ω*−*ω*_*c*_) represents the Dirac delta function at *ω* = *ω*_*c*_. From Eq. 1, it is straightforward to find that for a time-invariant metasurface with constant Γ(*t*), the reflected signal only contains information for the carrier frequency *ω*_*c*_. However, if the reflection coefficient Γ(*t*) turns out to be a periodic signal, it can be expressed as a linear combination of harmonically related complex exponentials

2 $$\Gamma (t) = \mathop {\sum}\limits_{k = - \infty }^{ + \infty } {a_ke^{jk\omega _0t}} = \mathop {\sum}\limits_{k = - \infty }^{ + \infty } {a_ke^{jk\frac{{2\pi }}{T}t}}$$

with the corresponding spectral expression for the reflected wave given as follows

3 $${\mathrm{E}}_r(\omega ) = 2\pi \mathop {\sum}\limits_{k = - \infty }^{ + \infty } {a_k{\mathrm{E}}_i(\omega - k\omega _0)}$$

in which *ω* = 2*π/T* represents the angular frequency determined by the function period *T* and *a*_*k*_ is the coefficient of the *k*^th^ harmonic component. Due to strong wave-matter interactions, a nonlinear phenomenon occurs as characterized by the emergence of numerous harmonics beside the fundamental components. Here, we focus on a specific scenario of a lossless metasurface with a reflection phase response described by the periodic square wave

4 $$\Gamma (t) = Ae^{j\left\{ {\varphi _1 + (\varphi _2 - \varphi _1)\mathop {\sum}\limits_{n = - \infty }^{ + \infty } {\left[ {\varepsilon \left( {t - nT} \right) - \varepsilon \left( {t - \frac{T}{2} - nT} \right)} \right]} } \right\}}$$

where *A* is the constant reflection amplitude, *φ*_1_,*φ*_2_ are the two phase states of the square wave and *ε*(*t* *−* *nT*) represents the unit step function shifted by *nT*. From Eqs. (2)–(4), the harmonic coefficient is given by:

5 $$a_k = \left\{ {\begin{array}{*{20}{l}} {A{\kern 1pt} {\mathrm{cos}}\frac{{\varphi _2 - \varphi _1}}{2}e^{j\frac{{\varphi _2 + \varphi _1}}{2}}} \hfill & {k = 0} \hfill \\ {\frac{{2A}}{{k\pi }}\sin \frac{{\varphi _2 - \varphi _1}}{2}e^{j\frac{{\varphi _2 + \varphi _1}}{2}}} \hfill & {k = \pm 1, \pm 3, \pm 5 \cdots} \hfill \\ 0 \hfill & {k = \pm 2, \pm 4, \pm 6 \cdots} \hfill \end{array}} \right.$$

The Fourier transform of Eq. (5) yields

6 $$\begin{array}{*{20}{l}} {{\mathrm{E}}_r(\omega )} \hfill & = \hfill & {2\pi A{\kern 1pt} {\mathrm{cos}}\frac{{\varphi _2 - \varphi _1}}{2}e^{j\frac{{\varphi _2 + \varphi _1}}{2}}{\mathrm{E}}_i(\omega )} \hfill \\ {} \hfill & {} \hfill & \hskip -20pt{ + {\kern 1pt} \mathop {\sum}\limits_{m = - \infty }^{ + \infty } {\frac{{4A}}{{2m - 1}}{\kern 1pt} {\mathrm{sin}}{\kern 1pt} \frac{{\varphi _2 - \varphi _1}}{2}e^{j\frac{{\varphi _2 + \varphi _1}}{2}}{\mathrm{E}}_i\left[ {\omega - (2m - 1)\omega _0} \right]}} \hfill \end{array}$$

**Fig. 7:**
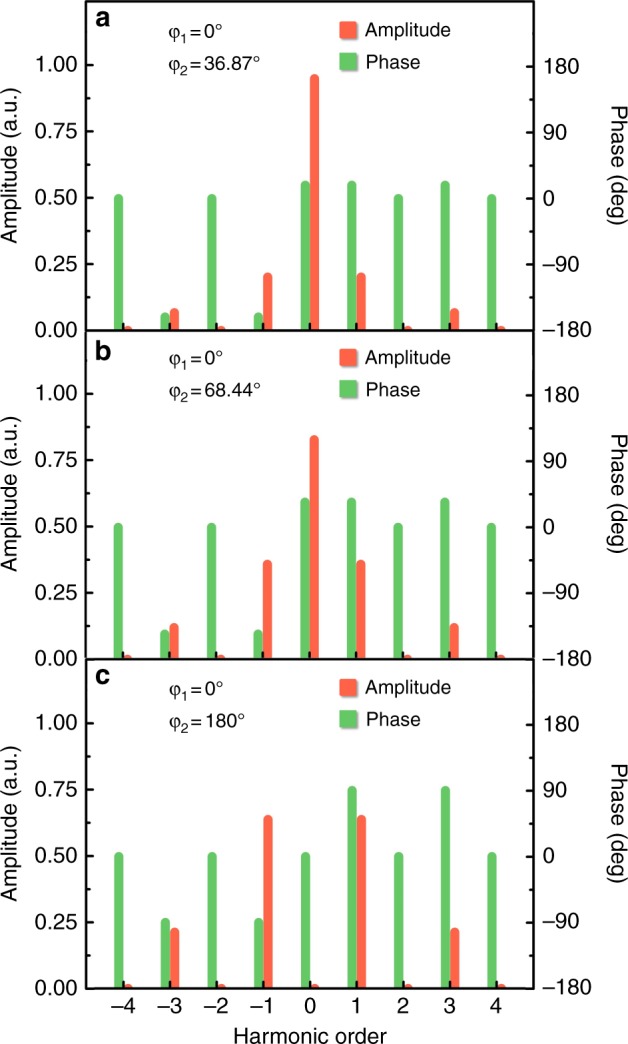
**Theoretical harmonic amplitude and phase distributions. a**–**c** The calculated harmonic amplitude and phase distributions under different combinations of φ_1_/φ_2_, which were set to 0°/36.87°, 0°/68.44° and 0°/180°, respectively

Only the synchronous component (*k* = 0) and odd harmonics are found to survive in the reflected waves, as can be understood in terms of the Fourier Transform of square waves. The envelope of the reflection spectra is highly associated with the phase pair (*φ*_1_,*φ*_2_), which in turn offers the opportunity to dynamically control the amplitude and phase distributions of high-order harmonics. To further illustrate this principle, the dependence of the reflection spectra on *φ*_1_ and *φ*_2_ is illustrated in Fig. 7. Three sets of phase combinations are taken into account with *φ*_1_/*φ*_2_ = 0°/36.87°, 0°/68.44° and 0°/180°, respectively. As the phase difference Δ*φ* = *φ*_1_ − *φ*_2_ gradually approaches 180°, the intensity of the synchronous component tends to be significantly suppressed, as shown in Fig. 7a–c. This phenomenon is consistent with the mathematical predictions in Eq. 6, where the first term that represents the synchronous component is proportional to the cosine function of Δ*φ*/2. Meanwhile, as a consequence, the harmonic energies are augmented, resulting in efficient frequency conversion. When the opposite phase requirement between *φ*_1_ and *φ*_2_ is satisfied, the synchronous components are totally cancelled under this circumstance, whereas the harmonics of the  ±1^st^ order reach the maximum amplitude with *a*_1_ = 0.6366. The calculated data in Fig. 7 were used to determine the contribution of the harmonics to overall reflection, which is dominated by the components *k* = ±1, accounting for 81.05 percent of the overall reflection energy in Fig. 7c. Such a proportion is especially favored for mixing applications, leading to new opportunities for up and down conversion with satisfactory efficiency in free space.

### Independent control of the harmonic amplitudes and phases by the time-domain digital coding metasurface

Until now, we have only considered the feasibility of harmonic generation through the time-domain digital coding metasurface. One drawback of the proposed scheme is the strong correlation between the harmonic amplitude and phase, thereby greatly hindering independent control of both parameters in practice. A possible recipe to overcome such limitation lies in the introduction of an additional time delay *t*_0_ to the time-varying reflection coefficient. Following the time shift property of a Fourier transform with $\Gamma (t - t_0) = {\cal F}^{ - 1}[e^{ - j\omega t_0}\Gamma (j\omega )]$, the time delay leads to an additional phase shift $e^{ - jk\omega _0t_0}$ for the *k*^th^ order harmonic, while maintaining an unchanged amplitude. After a more formal derivation, we obtain the spectral expression for the reflected wave in this case

7 $${{\mathrm{E}}_r(\omega )} = {2\pi A{\kern 1pt} {\mathrm{cos}}\frac{{\varphi _2 - \varphi _1}}{2}e^{j\frac{{\varphi _2 + \varphi _1}}{2}}{\mathrm{E}}_i(\omega )} \\ \hskip 10pt + {\kern 1pt} \mathop {\sum}\limits_{m = - \infty }^{ + \infty } \frac{{4A}}{{2m - 1}}{\kern 1pt} {\mathrm{sin}}{\kern 1pt} \frac{{\varphi _2 - \varphi _1}}{2}e^{j\left[ {\frac{{\varphi _2 + \varphi _1}}{2} - (2m - 1)\omega _0t_0} \right]} \\ \hskip 20pt {\mathrm{E}}_i\left[ {\omega - (2m - 1)\omega _0} \right]$$

Specifically, the amplitude and phase of the *k*^th^ order harmonic are rewritten as

$${{\mathrm{E}}_r(k\omega _0 + \omega _c) = \left| {\mathrm{A}} \right|\angle \Phi }$$

8 $${ = \left\{ {\begin{array}{*{20}{l}} {\left| {A{\kern 1pt} {\mathrm{cos}}{\kern 1pt} \frac{{\varphi _2 - \varphi _1}}{2}} \right|e^{j\left\{ {\frac{{\varphi _2 + \varphi _1}}{2} + \pi \left[ {\varepsilon \left( {\frac{{\varphi _2 - \varphi _1 - \pi }}{2}} \right) + \varepsilon \left( {\frac{{\varphi _1 - \varphi _2 - \pi }}{2}} \right)} \right]} \right\}}} \hfill & {k = 0} \hfill \\ {\left| {\frac{{2A}}{{k\pi }}{\kern 1pt} {\mathrm{sin}}{\kern 1pt} \frac{{\varphi _2 - \varphi _1}}{2}} \right|e^{j\left\{ {\frac{{\varphi _2 + \varphi _1}}{2} - \pi \left[ {\varepsilon (\varphi _2 - \varphi _1) + \varepsilon (k)} \right] - k\omega _0t_0} \right\}}} \hfill & {k = \pm 1, \pm 3, \pm 5 \cdots} \hfill \\ 0 \hfill & {k = \pm 2, \pm 4, \pm 6 \cdots} \hfill \end{array}} \right.}$$

The extra phase shift provides new degrees of freedom to control the propagation features of high-order harmonics by breaking the constraint between their amplitude and phase, making it possible to realize independent regulation of both parameters, which is hard to achieve using conventional methods. In fact, Eq. (8) offers a promising route to tailor the response of reflected harmonics via the combination of three parameters *φ*_1_,*φ*_2_, and *t*_0_, consequently pushing the intriguing functionalities accomplished by traditional metasurfaces into the nonlinear regime.

The concept of a digital coding metasurface arises from a simple analogy with digital circuits, where a finite number of element types are employed to manipulate electromagnetic waves. For a 1-bit metasurface, only two kinds of elements (named as ‘0’ and ‘1’ element) with opposite phase responses are utilized to constitute the entire structure. By alternating the spatial alignment of both elements, the metasurface demonstrates a powerful capability to reach a broad range of functionalities such as anomalous beam reflections and random diffusion. Such ideas can be easily extended to design of the metasurface in the time domain. For example, given the phase states *φ*_1_,*φ*_2_ in Eq. (8), it is easy to construct a ‘0’ and ‘1’ element by inserting a time delay *t*_0_ for the *k*^th^ order harmonic. Here, we choose *t*_0_ = 0 and *T*/2 respectively, in which *T* is the period of the square function in Eq. (4). As a quantitative illustration of nonlinear control, we considered a scenario where a plane wave is illuminated normally at *f*_c_ = 3.7GHz upon the 1-bit time-domain digital coding metasurface. In this case, we use eight columns of meta-atoms in total with a unit period P = 33 mm. The repeating frequency of the square wave was *f*_0_ = 156.25 kHz. Figure 5a, c, e shows the calculated E-plane scattering pattern for the +1^st^ order harmonic under different coding sequences ‘0000…’, ‘00001111…’, and ‘00110011…’. From the antenna theory, the scattering pattern is shown to hold as a Fourier transform pair with the phase distributions within the metasurface. Therefore, the scattering pattern can be directly inferred from the coding sequences of the binary elements through mathematical calculations. The backward scattering beam is split into two symmetric beams directed along either side, with the tilting angle gradually increased, as shown in Fig. 5a, c, e. Such scattering behavior is closely associated with the introduction of the parallel wave-vector that is imposed onto the reflected wave with the change of spatial arrangement for the ‘0’ and ‘1’ elements.

In addition, the red, blue and green lines in Fig. 5a, c, e demonstrate the dependence of the scattering magnitude of the  +1^st^ order harmonic under different phase combinations of *φ*_1_/*φ*_2_ as 0°/180°, 0°/68.44° and 0°/36.87°, respectively. The scattering pattern undergoes a uniform attenuation in its amplitude by 0, 5, and 10 dB with the change in phase pair.

One important concern related to the metasurface is the conversion efficiency from the fundamental harmonic to high-order harmonics. To address this issue, we compare the measured and theoretical conversion efficiency of the −4^th^ to  +4^th^ order harmonics in Tables 2 and 3, with *V*_1_/*V*_2_(*φ*_1_/*φ*_2_)= V/12 V(0°/180°), *T* = 6.4 μs and *V*_1_/*V*_2_(*φ*_1_/*φ*_2_)= V/18 V (0°/68.44°), *T* = 6.4 μs, respectively. The efficiency is obtained from the energy ratio between the harmonic and incident wave. From Table 2, when *φ*_1_ and *φ*_2_ are out of phase, the measured fundamental wave occupies 3.49% of the total energy, indicating that 96.51% of the incident power is converted to high-order harmonics. The majority of the energy is assigned to ±1^st^ harmonics, as expected. In general, the theoretical and measured results for each harmonic are in good accordance. In the measurement, when *φ*_1_ − *φ*_2_ = 68.44°, only 30% of the incident energy is converted into high-order harmonics, consistent with the theoretical prediction in Table 3.

**Table 2:** Measured and theoretical conversion efficiency when *V*_1_/*V*_2_(φ_1_/φ_2_) = 0 V/12 V (0°/180°) with *T* = 6.4 μs

Harmonic order	Measured *V*_1_/*V*_2_ = 0 V/12 V	Theoretical *φ*_1_/*φ*_2_ = 0°/180°	Error
−4^th^	0.08%	0%	0.08%
−3^rd^	3.14%	4.50%	−1.36%
−2^nd^	0.08%	0%	0.08%
−1^st^	34.93%	40.53%	−5.60%
0^th^	3.49%	0%	3.49%
+1^st^	35.87%	40.53%	−4.66%
+2^nd^	0.09%	0%	0.09%
+3^rd^	3.31%	4.50%	−1.19%
+4^th^	0.10%	0%	0.10%

**Table 3:** Measured and theoretical conversion efficiency when *V*_1_/*V*_2_(φ_1_/φ_2_) = 9 V/18 V (0°/68.44°) with *T* = 6.4 μs

Harmonic order	Measured *V*_1_/*V*_2_ = 9 V/18 V	Theoretical *φ*_1_/*φ*_2_ = 0°/68.44°	Error
−4^th^	0.01%	0%	0.01%
−3^rd^	0.87%	1.42%	−0.55%
−2^nd^	0.01%	0%	0.01%
−1^st^	7.83%	12.82%	−4.99%
0^th^	70.90%	68.38%	2.52%
+1^st^	7.43%	12.82%	−5.39%
+2^nd^	0.01%	0%	0.01%
+3^rd^	0.81%	1.42%	−0.61%
+4^th^	0.01%	0%	0.01%
